# Is repeated pulmonary metastasectomy justified?

**DOI:** 10.1007/s10585-020-10056-w

**Published:** 2020-09-13

**Authors:** Céline Forster, Amaya Ojanguren, Jean Yannis Perentes, Matthieu Zellweger, Sara Federici, Thorsten Krueger, Etienne Abdelnour-Berchtold, Michel Gonzalez

**Affiliations:** 1 Lausanne University Hospital (CHUV), Rue du Bugnon 46, 1011 Lausanne, Switzerland; 2 Faculty of Biology and Medicine, University of Lausanne (UNIL), Rue du Bugnon 21, 1011 Lausanne, Switzerland

**Keywords:** Pulmonary metastases, Pulmonary metastasectomy, VATS, Repeat metastasectomy

## Abstract

Recurrence after pulmonary metastasectomy (PM) is frequent, but it is unclear to whom repeated pulmonary metastasectomy (RPM) offers highest benefits. Retrospective analysis of oncological and post-operative outcomes of consecutive patients who underwent PM from 2003 to 2018. Overall survival (OS) and disease-free interval (DFI) were calculated. Cox regression was used to identify variables influencing OS and DFI. In total, 264 patients (female/male: 114/150; median age: 62 years) underwent PM for colorectal cancer (32%), sarcoma (19%), melanoma (16%) and other primary tumors (33%). Pulmonary metastasectomy was approached by video-assisted thoracic surgery (VATS) in 73% and pulmonary resection was realized by non-anatomical resection in 76% of cases. The overall median follow-up time was 33 months (IQR 16–56 months) and overall 5-year survival rate was 62%. Local or distant recurrences were observed in 172 patients (65%) and RPM could be performed in 66 patients (25%) for a total of 116 procedures. RPM was realized by VATS in 49% and pulmonary resection by wedge in 77% of cases. In RPM patients, the 5-year survival rate after first PM was 79%. Post-operative cardio-pulmonary complication rate (13% vs. 12%; p = 0.8) and median length of stay (4 vs. 5 days; p = 0.2) were not statistically different between first PM and RPM. Colorectal cancer (HR 0.56), metachronous metastasis (HR 0.48) and RPM (HR 0.5) were associated with better survival. In conclusion, our results suggest that RPM offers favorable survival rates without increasing post-operative morbidity.

## Introduction

Pulmonary metastases are frequently encountered in patients with solid primary tumors [1–4]. Pulmonary metastasectomy (PM) has become an accepted part of a multidisciplinary treatment [5], if (i) the primary tumor is controlled, (ii) there is no other extra-thoracic metastasis and (iii) the patients can tolerate the surgery [6]. In addition, in light of recent improvements in surgical and radiological technologies, such as the Video-Assisted Thoracic Surgery (VATS) approach and the 1-mm thin slice CT scan, PM has become technically easier and morbidity and mortality rates have decreased [7, 8].

However, recurrences after PM occur in more than 50% of patients, making some of them potential candidates for a repeated PM (RPM) [1, 9, 10]. The indications for RPM are controversial, particularly since systemic and radiological therapies, such as chemotherapy, immunotherapy and stereotactic body radiotherapy are continuously improving [11, 12]. Moreover, repeated surgeries are more technically challenging due to pleural adhesions and patients are prone to higher post-operative morbidity rates due to their lower pulmonary capacity [13, 14]. Yet, some series have reported encouraging results after RPM with increased 5-year survival rates in patients undergoing RPM (58–79%) compared to those who did not undergo RPM (24–52%) [14–16], and PM itself may also provide useful information on the nature of a nodule that might avoid unnecessary treatment in case of benign lesion.

The aim of this study was to compare post-operative outcomes and survival prognosis of first PM and RPM. Survival prognostic and recurrence risk factors were also analyzed.

## Material and methods

### Patient selection and study design

We reviewed retrospectively all patients undergoing PM or RPM in our institution between July 2003 and November 2018. The selection criteria were based on currently accepted guidelines [5]: (i) the metastases are potentially resectable; (ii) the primary tumor is under control at its original location; (iii) presence of other, extra-thoracic metastasis is excluded or fully resectable prior to PM/RPM; (iv) the patient is expected to have sufficient respiratory functions after surgery; (v) surgery is the only or the best therapeutic option. We excluded from this study all patients undergoing a diagnostic procedure. The inclusion and exclusion criteria were the same for PM and for RPM.

The study was approved by the Local Ethics Committee (N° 2019-02474) and individual consent was waived.

### Data collection

Patients’ medical records were reviewed individually and data was extracted retrospectively from our database. Selected relevant data included patient demographics, comorbidities, oncological characteristics and surgical profile. We compiled post-operative outcomes including cardiopulmonary complications for up to 30 days after surgery, readmission rates, re-operation rates, recurrences and RPM characteristics. We analysed overall survival (OS) and disease-free interval (DFI). The time interval between the resection of the primary tumor and the first PM was defined as DFI1 and the time interval between first PM and cancer recurrence was defined as DFI2. We called “synchronous” the pulmonary metastases identified at the time of the primary tumor resection and “metachronous” those identified after initial treatment of primary tumor. Bilateral synchronous metastases were defined as a single entity and their resection not considered an RPM even if it had to happen in separate surgeries. Pulmonary metastasis recurrence was defined as the apparition of a new pulmonary metastasis after initial disease control.

### Operation and follow-up

Before surgery, all cases were individually discussed in an interdisciplinary tumor board to assess the indications for surgery. Surgical procedures (PM or RPM) were performed under general anaesthesia with double-lumen intubation and single-lung ventilation. When necessary, a hook wire device was pre-operatively placed (under CT-scan control) to help intraoperative localization of pulmonary metastases. We selected the most adequate surgical approach based on each patient’s history and on the characteristics of the pulmonary metastases (size, localisation and number). A VATS approach was generally preferred when the number of pulmonary metastases was < 3, each pulmonary metastasis was peripherally located and amenable to wedge-resection or, in case of central lesions, anatomical resection was possible. Additionally, the size of the lesion was determined to assess if it could be removed through the utility incision without rib retractor (normally less than 5 cm). If these criteria were not met or when anatomical resection was required by VATS but lesser resection was possible by thoracotomy, we generally selected a standard postero-lateral thoracotomy based on our policy to spare pulmonary parenchyma to the maximal possible extent. For VATS-mediated surgeries, we used a standard three-port anterior approach. Lymph node dissection was performed when there was a preoperative radiological suspicion of lymph node metastases or when an anatomical resection was chosen as the surgical option. All specimens were extracted through a protective bag. Our main objective was to achieve complete resection with healthy margins. All specimens were examined by the surgeon immediately after their extraction for clarity of the margins and, in case of doubt, a frozen-section analysis was carried out for histological confirmation. An interdisciplinary tumor board discussed the final histopathological status to assess the indications for an adjuvant treatment. The routine follow-up for all PM/RPM patients involved a thoraco-abdominal CT-scan at regular time points post-surgery (3, 6, 12, 18 and 24 months and then yearly).

### Statistical analysis

Continuous data are presented as median (interquartile range (IQR)) and categorical data as frequency with percentage. We compared patients’ characteristics and post-operative outcomes between first PM and RPM. Continuous variables were tested by the unpaired Student’s *t*-test whereas categorical variables were tested by the χ^2^ test. The OS and DFI were calculated using the Kaplan–Meier formula and compared with a log-rank test. A Cox regression univariate analysis was performed to identify potential prognostic factors of recurrence and survival. We also compared the post-operative outcomes with respect to cardio-pulmonary complications and length of stay between the first metastasectomy and with the total number of repeated resections. A two-tailed hypothesis was used and significance accepted if p < 0.05. All statistical analyses were performed using the Stata version 14 software (StataCorp, Texas USA).

## Results

A total of 264 patients (114 female/150 male) underwent PM in our institution between 2003 and 2018 in the context of colorectal (32.2%), sarcoma (18.9%), melanoma (15.9%) and other primary tumors (33%). Patient characteristics are shown in Table 1. Pulmonary metastases were single or multiple (61.4% vs. 38.6% of cases), and unilateral or bilateral (78.4% vs. 21.6% of cases). Other non-pulmonary metastases were diagnosed and treated by radiotherapy or surgery in 85 patients (32.3%), with 37 of them (14% of the studied patients) in the liver, 22 (8.3%) in the lymph nodes, 5 (1.9%) in the brain and 21 (8%) in other localisations. More than half of the patients (55.3%) underwent chemotherapy before first PM. The median DFI1 was 25 months (IQR 11–49 months).

**Table 1:** Patient characteristics

	First PM
Number of patients	264
Sex	
Female	114 (43.2%)
Male	150 (56.8%)
Age (years) (median)	62 [IQR 51–69.5]
Comorbidities	
Cardiopathy	19 (7.2%)
High blood pressure	92 (34.9%)
Pulmonary disease	20 (7.6%)
Tobacco exposure	75 (28.4%)
Diabetes	35 (13.3%)
Renal failure	15 (5.7%)
Immunosuppression	10 (3.8%)
Primary tumor	
Colorectal	85 (32.2%)
Sarcoma	50 (18.9%)
Melanoma	42 (15.9%)
Head and neck	21 (8%)
Upper gastrointestinal tract	16 (6.1%)
Urological	14 (5.3%)
Breast	11 (4.2%)
Gynecological	7 (2.7%)
Thyroid	6 (2.3%)
Testicular	5 (1.9%)
Other	7 (2.7%)
Pulmonary metastases	
Single	162 (61.4%)
Multiple	102 (38.6%)
Unilateral	207 (78.4%)
Bilateral	57 (21.6%)
Synchronous	30 (11.4%)
Size (mm) (median)	11 [IQR 7–16]
Margins (mm) (median)	5 [IQR 2–10]
R0	254 (96.2%)
R1	9 (3.4%)
R2	1 (0.4%)
Lymph node involvement	16 (6.1%)

*PM* pulmonary metastasectomy; cardiopathy (defined as the presence of ischemic events in the past, cardiac insufficiency, arrhythmia or aortic aneurysm); high blood pressure (defined as systolic arterial pressure > 140 mmHg); pulmonary disease (defined as the presence of chronic obstructive pulmonary disease, fibrosis, pulmonary hypertension or sleep apnoea syndrome); diabetes (defined as fasting plasma glucose > 7 mmol/l); renal failure (defined as glomerular filtration rate < 30 ml/min/1.73m^2^); R0 (defined as the absence of cancer cells seen microscopically at the tumor site); R1 (defined as the presence of cancer cells microscopically at the tumor site); R2 (defined as macroscopic residual tumor at cancer site or regional lymph nodes)

The PMs were performed by VATS in 193 (73.1%) and thoracotomy in 71 (26.9%) patients. Surgical characteristics are shown in Table 2. Two conversions from VATS to thoracotomy were necessary because of centrally located lesions non-resectable by VATS. Most of the surgeries were achieved by wedge resection (75.8%). Segmentectomies and lobectomies were performed in 20 (7.6%) and 42 (15.9%) patients, respectively. Two patients needed a pneumonectomy, one because of a centrally located metastasis infiltrating the pulmonary artery and the other one because of a post-radiation stenosis of the left main bronchus. Mediastinal lymph node dissection was undertaken in 57 (21.6%) patients. The median duration of hospital stays and of drainage was 4 days (IQR 3–8 days) and 1 day (IQR 1–3 days), respectively.

**Table 2:** Surgical characteristics of first pulmonary metastasectomy (PM) and repeated pulmonary metastasectomy (RPM)

	First PM (n = 264)	RPM (n = 116)	P-value
Surgical characteristics			
VATS	193 (73.1%)	57 (49.1%)	< 0.0001
Thoracotomy	71 (26.9%)	59 (50.9%)	< 0.0001
Wedge resection	200 (75.8%)	89 (76.7%)	0.84
Segmentectomy	20 (7.6%)	13 (11.2%)	0.25
Lobectomy	42 (15.9%)	12 (10.3%)	0.16
Pneumonectomy	2 (0.8%)	2 (1.7%)	0.41
Mediastinal lymph nodes dissection	57 (21.6%)	14 (12.1%)	0.03
Post-operative outcomes			
Overall 30-day mortality	0	0	N/A
Cardio-pulmonary complications (30-day)	34 (12.9%)	14 (12.1%)	0.83
Duration of drainage (days) (median)	1 [IQR 1–3]	2 [IQR 1–4]	0.01
Duration of hospital stay (days) (median)	4 [IQR 3–8]	5 [IQR 3–8]	0.21
Readmission (30-day)	5 (1.9%)	0	N/A
Re-operation (30-day)	4 (1.5%)	0	N/A

*PM* pulmonary metastasectomy; *RPM* repeated pulmonary metastasectomy; *VATS* Video-Assisted Thoracic Surgery

Readmission was necessary in 5 patients (1.9%) for pleural effusion (n = 2), febrile neutropenia (n = 1), chest wall hematoma requiring surgical revision (n = 1) and additional resection for R1 resection (n = 1). Two more patients were re-operated during the 30-day post-operative period for prolonged air leak (> 4 days) with an aerostasis achieved by VATS, adding up to a re-operation rate of 1.5% (n = 4). Overall 30-day morbidity was 15.5% and 12.9% were cardio-pulmonary complications. There was no 30-day post-operative mortality.

During the follow-up period (median 33 months (IQR 16–56 months)), 30 (11.4%) patients were lost to follow-up and 172 (65.2%) patients presented at least one recurrence (46 in the lung only (26.7%); 38 distantly (22.1%); 87 in both localisations (50.6%)). The median DFI2 was 10 months (IQR 4–29 months). Only 66 of the 172 patients who developed recurrence (38%) underwent an RPM. Of those 66 patients, 38 presented a pulmonary recurrence on the operated side (58.6%), 17 on the contralateral side (25.8%) and 11 on both sides (15.7%). A first RPM was performed in 66 patients (25% of the original population of 264 patients), a second in 33 (12.5%), a third in 14 (5.3%), a fourth in 2 (0.8%) and fifth in one patient (0.4%) for a total of 116 RPM procedures. The total RPM procedures characteristics are shown in Table 2. For the 116 RPM procedures, VATS approach was preferred in 57 cases (49.1% of RPMs) and wedge resections performed in 76.7% of cases. The 30-day post-operative cardio-pulmonary complication rate was similar between first PM and RPM (12.9% vs. 12.1%; p = 0.8). There was no readmission, re-operation or 30-day post-operative mortality after RPM. The median duration of hospital stay was not statistically different between PM and RPM (4 vs. 5 days; p = 0.21). The median duration of drainage was significantly longer in the RPM group (2 vs. 1 day; p = 0.01).

On univariate analysis, we identified four risk factors of recurrence (Table 3): age < 70 years (HR 1.80, 95% CI 1.21–2.68, p = 0.004), non-colorectal tumors (HR 1.48, 95% CI 1.06–2.07, p = 0.02), previous extra-thoracic metastases (HR 1.57, 95% CI 1.15–2.16, p = 0.005) and multiple pulmonary metastases (HR 1.59, 95% CI 1.17–2.16, p = 0.003).

**Table 3:** Univariate analysis of risk factors of recurrence after first pulmonary metastasectomy (PM)

Variables	Univariate		
	HR	95% CI	p value
Female sex	0.93	0.69–1.27	0.67
Age < 70 years	1.80	1.21–2.68	0.004*
Non-colorectal tumor	1.48	1.06–2.07	0.02*
Previous extra-thoracic metastases	1.57	1.15–2.16	0.005*
Chemotherapy before first PM	1.04	0.77–1.42	0.79
DFI1 < 12 months	1.25	0.89–1.76	0.19
Metachronous metastases	0.86	0.52–1.43	0.57
Multiple pulmonary metastases	1.59	1.17–2.16	0.003*
Unilateral pulmonary metastases	0.89	0.62–1.26	0.5
VATS	0.81	0.58–1.14	0.22
Wedge resection	1.25	0.86–1.83	0.24
Margins of the pulmonary metastasis < 5 mm	1.31	0.96–1.79	0.08
Size of the largest pulmonary metastasis < 20 mm	0.88	0.60–1.29	0.5
Mediastinal lymph nodes involvement	1.36	0.69–2.67	0.37

*HR* hazard ratio; *CI* confidence interval; *PM* pulmonary metastasectomy; *DFI1* disease-free interval between primary tumour resection and first pulmonary metastasectomy; *VATS* Video-Assisted Thoracic Surgery

**Fig. 1:**
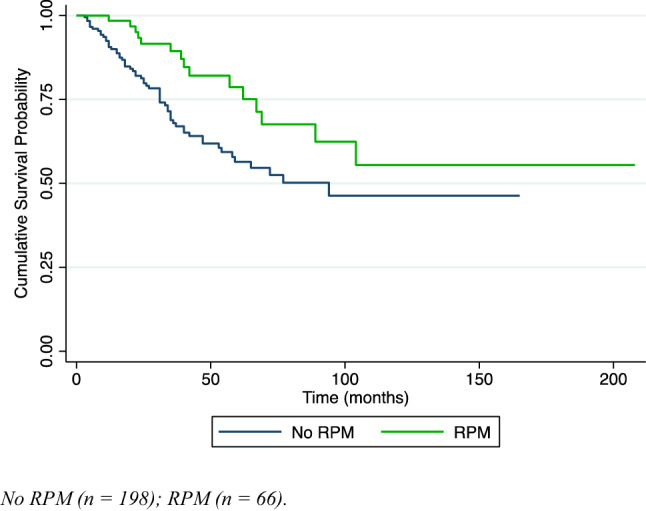
Kaplan–Meier curves of overall survival after first pulmonary metastasectomy (PM) in patients without (no RPM) and with repeated pulmonary metastasectomy (RPM)

The overall 5-year survival rate after first PM was 62% (calculated on the entire population, n = 264). In patients who underwent one PM only (n = 198), the 5-year survival rate was 56.4%. For the group of patients who underwent one or several RPMs (n = 66), the 5-year survival rate was 79%, statistically significantly higher than after the first PM or one PM only (log rank test; p = 0.014) (see Fig. 1). The overall median survival after the second PM was 31.5 months. The prognostic factors of prolonged survival (Table 4) were colorectal cancer (HR 0.56, 95% CI 0.33–0.95, p = 0.03), metachronous metastases (HR 0.48, 95% CI 0.25–0.95, p = 0.04) and RPM (HR 0.50, 95% CI 0.28–0.87, p = 0.02).

**Table 4:** Univariate analysis of factors associated with survival after first pulmonary metastasectomy (PM)

Variables	Univariate		
	HR	95% CI	p value
Female sex	0.98	0.62–1.55	0.93
Age < 70 years	1.14	0.63–2.05	0.66
Colorectal tumor	0.56	0.33–0.95	0.03*
Previous extra-thoracic metastases	1.19	0.74–1.90	0.48
Chemotherapy before first PM	1.21	0.77–1.91	0.41
DFI1 < 12 months	1.48	0.89–2.45	0.13
Metachronous metastases	0.48	0.25–0.95	0.04*
Solitary pulmonary metastasis	0.74	0.47–1.17	0.20
Unilateral pulmonary metastasis	0.81	0.48–1.34	0.41
VATS	0.75	0.46–1.23	0.26
Wedge resection	1.27	0.67–2.41	0.47
Margins of the pulmonary metastasis < 5 mm	1.14	0.72–1.81	0.56
Size of the largest pulmonary metastasis < 20 mm	0.97	0.53–1.76	0.92
Mediastinal lymph nodes involvement	1.68	0.61–4.64	0.32
RPM	0.50	0.28–0.87	0.02*

*HR* hazard ratio; *CI* confidence interval; *PM* pulmonary metastasectomy; *DFI1* disease-free interval between primary tumour resection and first pulmonary metastasectomy; *VATS* Video-Assisted Thoracic Surgery; RPM: repeated pulmonary metastasectomy

## Discussion

We report the post-operative outcomes of 264 patients undergoing PM or RPM. Recurrences were observed in 172 (65.2%) patients and 66 of them (25%) underwent at least one RPM procedure for a total of 116 RPM procedures. The 5-year survival rate was significantly higher in the patients who underwent an RPM compared to those who underwent one PM only (79% vs. 56.4%; p = 0.014).

We identified three factors associated with prolonged survival for PM patients: colorectal tumors, metachronous metastases and RPM. Some other prognostic factors of survival have been described in the literature, but were not identified as statistically significant in our series: DFI1; completeness of resection; presence of lymph node metastases; number and size of pulmonary metastases; female sex [2]. The primary tumor histology has been identified as a prognostic factor in many studies [1, 2, 17, 18]. The International Registry of Lung Metastases reported a better survival rate for patients with germ cell tumors (68% at 5 years), followed by epithelial tumors (37% at 5 years), sarcomas (31% at 5 years) and melanoma (21% at 5 years) [1]. We identified the colorectal cancer patients to have the best survival prognosis. In 2018, Hirai et al. retrospectively reviewed 106 patients undergoing PM in the context of various primary tumors histology [18]. They also found a better survival prognosis for patients with colorectal carcinoma (p = 0.003). Similarly, the International Registry of Lung Metastases reported a higher rate of recurrences for sarcomas and melanoma (64%) than for epithelial (46%) or germ cell (26%) tumors [1]. This means that the biology of the primary tumor is an important factor associated with recurrence.

We also identified the metachronous development of metastases (HR 0.48, 95% CI 0.25–0.95, p = 0.04) as prognostic factor of better survival. In their meta-analysis including 1447 patients with renal cancer undergoing PM, Zhao et al. identified the synchronous development of metastases as being a poor prognostic factor (HR 2.49, 95% CI 1.46–4.24, p = 0.001) [19]. In fact, the earlier the pulmonary metastases are diagnosed, the more aggressive the primary tumor can be assumed to be. Other prognostic factors of recurrence have been described, such as DFI shorter than 1-year, female sex and atypical resection, which were not found to be statistically relevant in our study [10, 15, 20].

The third factor that we identified to be associated with prolonged survival was RPM. In case of pulmonary recurrence, we performed RPM using to the same inclusion/exclusion criteria as for the first PM [5]. The 5-year survival rate after first PM in case of RPM was statistically higher than without RPM (79% vs. 56.4%; p = 0.014). The median survival after second PM was 31.5 months. Some other series similarly reported a survival advantage for patients who underwent RPM compared to those who did not [15, 16, 21], and some authors openly doubt that PM might even provide a survival advantage [22]. In their retrospective study including 238 patients with pulmonary metastases from colorectal cancer, Sponholz et al. found a 5-year survival rate of 75% for the patients who underwent RPM (a figure similar to the one we are reporting) and 24% for patients who did not (p < 0.001) [15]. Same results were observed by Salah et al. and Welter et al., with a 5-year survival rate of 40–50% for patients undergoing a single PM and 55–60% for patients undergoing RPM [16, 21]. On the other hand, in the series of Menna et al. including 173 patients, this difference was not statistically significant (p = 0.659) [14]. Those conflicting results are yet to be analyzed in a case-matched controlled trial comparing the survival rate after first PM in patients undergoing RPM and in patients with no repeated surgery, but they tend to point towards a tendency that might be of highest interest to the patients and their families.

Despite the improved survival prognosis after PM, up to 50% of patients present a recurrence, with 20–40% of pulmonary recurrences [1, 9, 10, 16]. In such cases however, RPMs are usually performed in only 20% of such patients [1, 9, 10, 16, 21]. This low percentage is explained by the decreasing patient’s tolerance to pulmonary resection and the diffuse metastatic spreading of the primary tumors, warranting exclusion of the patients from consideration for an RPM [1, 5, 6, 14, 21]. In our study, the recurrence and RPM rates were concordant with those reported in the literature (65.15% and 25%, respectively). We identified four risk factors of recurrence (age < 70 years, non-colorectal tumors, previous extra-thoracic metastases and multiple pulmonary metastases). The younger age has already been described as a prognostic factor of recurrence by Onaitis et al. (HR 0.71, 95% CI 0.51–0.99, p = 0.04) [20]. It might appear counter-intuitive that younger age could represent a risk factor of recurrence, but these authors posited the hypothesis that a selection bias was involved (younger patients are offered more aggressive treatments, sustain the treatment better, thus have *in fine* more time to develop recurrences). In addition, it is a fair assumption that aggressive primary tumors, which generally grow very quickly and tend to metastasize faster, are more likely to be treated in younger patients who have a longer survival prognosis to begin with. This might explain why the presence of prior extra-thoracic metastases and the multiplicity of pulmonary metastases are reported to be prognostic factors of recurrence [10, 15, 20].

Post-operative cardio-pulmonary morbidity was acceptable after both first PM and subsequent RPMs, showing similar rates (12.9% and 12.1%; p = 0.8). This is aligned with results reported by others [14, 21]. Menna et al. reported a post-operative morbidity rate of 11.3% after RPM and 18.3% after first PM, with no statistically significant difference [14]. We also observed that the patients undergoing PM are usually aged (median 62 years in our study) and present several comorbidities, making them at risk for potential postoperative morbidity. The Spanish prospective cohort of 532 colorectal cancer patients undergoing PM reported an overall morbidity of 15.6% and mortality of 0.4% [8]. These low morbidity rates can be explained by our high percentage of minimally invasive approach (VATS) and parenchyma-sparing techniques (wedge or segmentectomy) used in most PM and RPM procedures. Thus, PM can be easily repeated with little chest wall trauma and pleural adhesions. In their study on 46 patients undergoing RPM, Kondo et al. demonstrated that a previous VATS procedure was associated with fewer pleural adhesions during the redo surgery than a previous thoracotomy [23]. Moreover, the VATS procedures showed significantly shorter operating times and durations of drainage and lower intraoperative bleeding (p < 0.05). In our experience, we observed that RPM procedures were better accepted by patients and referent physicians if the first PM was performed by VATS, likely due to its low morbidity rate [7, 24].

In addition, several recent reports have reported that the VATS approach is as efficient as thoracotomy in terms of survival, recurrence and oncological results [9, 23, 25, 26]. In the retrospective case-matched study of Chao et al. including colorectal cancer patients undergoing PM, thoracotomy and VATS groups had similar recurrence rates (54 vs. 40%; p = 0.23) and 5-year overall survival rates (43% vs. 51%; p = 0.21) [9]. A meta-analysis including colorectal cancer and sarcoma patients found the same results in terms of recurrence-free survival rate (HR 0.86, 95% CI 0.69–1.08, p = 0.2) and overall survival rate (HR 0.78, 95% CI 0.59–1.03, p = 0.075) [26]. Despite described favorable survival prognosis for the anatomical resections (lobectomy and pneumonectomy) in some colorectal cancer series, we normally reserve these types of resection for large, centrally located pulmonary metastases of colorectal cancer origin [27, 28]. Our policy is to spare pulmonary parenchyma as much as possible, in agreement with current expert views on this matter [5, 29]. For centrally located lesions, we consider that segmentectomies may be a good parenchyma-sparing alternative with an acceptable morbidity rate even if performed by VATS [30]. In our study, we did not observe differences in term of recurrence or survival based on the extent of resection (wedge vs. anatomical resections).

The main limitation of our study is the retrospective, single center design. Another limitation is the absence of some pre-operative information, such as the systemic therapy regime and the primary tumor stage, both of which can have an influence on patient’s prognosis. Moreover, the patient inclusion spans a long time interval (2003–2018). During this period, there have been some innovations in patients’ management, which might have affected the standard of care that patients received. We did not, however, stratify our analyses by periods within the time of study because most of our patients were operated in the last years of the inclusion period and we accepted that this was of minimal impact on the statistical analysis.

Despite these limitations, our study includes a large collective of patients with pulmonary metastases and a complete follow-up. Finally, any patient undergoing an RPM is by definition someone who survived the previous PM(s). This of course does by no means disqualify the validity of RPM as a treatment method, but it induces (i) a clear selection bias, (ii) a distortion in the causality that cannot be corrected and (iii) a mathematical consequence that for any subsequent RPM, the number of patients will necessarily be smaller than for the previous PM. These elements do not question or weaken our observations or conclusions, but they must be borne in mind when accepting our conclusions that these must be considered with some degree of caution.

## Conclusions

In conclusion, this study showed that RPM is justified for selected patients with resectable recurrent pulmonary metastases who can tolerate a repeated surgery. An RPM procedure is feasible by VATS approach and provides good survival rate without increasing post-operative morbidity.

## Data Availability

Data is available upon request to the authors.

## Code availability

Commercial and open source, publicly-available software only.
